# A Genome-Wide Association Study to Detect QTL for Commercially Important Traits in Swiss Large White Boars

**DOI:** 10.1371/journal.pone.0055951

**Published:** 2013-02-05

**Authors:** Doreen Becker, Klaus Wimmers, Henning Luther, Andreas Hofer, Tosso Leeb

**Affiliations:** 1 Institute of Genetics, Vetsuisse Faculty, University of Bern, Bern, Switzerland; 2 Leibniz Institute for Farm Animal Biology, Dummerstorf, Germany; 3 SUISAG, Sempach, Switzerland; University of Queensland, Australia

## Abstract

The improvement of meat quality and production traits has high priority in the pork industry. Many of these traits show a low to moderate heritability and are difficult and expensive to measure. Their improvement by targeted breeding programs is challenging and requires knowledge of the genetic and molecular background. For this study we genotyped 192 artificial insemination boars of a commercial line derived from the Swiss Large White breed using the PorcineSNP60 BeadChip with 62,163 evenly spaced SNPs across the pig genome. We obtained 26 estimated breeding values (EBVs) for various traits including exterior, meat quality, reproduction, and production. The subsequent genome-wide association analysis allowed us to identify four QTL with suggestive significance for three of these traits (p-values ranging from 4.99×10^−6^ to 2.73×10^−5^). Single QTL for the EBVs pH one hour post mortem (pH1) and carcass length were on pig chromosome (SSC) 14 and SSC 2, respectively. Two QTL for the EBV rear view hind legs were on SSC 10 and SSC 16.

## Introduction

One of the most challenging tasks in pork production is the improvement of traits with low heritability [1]–[3]. Most commercially important traits are complex and influenced by multiple interacting factors including genetics and environment. The observed albeit low heritabilities suggest that these traits could be successfully improved by selection. However, the cost and difficulty to obtain direct measurements are limiting the improvements of these traits. In addition, the estimation of many breeding values relies only on the phenotypes of relatives, which limits their accuracy. Therefore, these traits are ideal candidates for the application of molecular genetic tools in future breeding programs. The identification of genes and polymorphisms associated with commercially important traits can provide useful markers for the selection of genetically superior animals. Knowledge of the genetic and molecular background is required to accelerate the genetic improvement.

Since the first QTL genome scan in pigs was reported [4] numerous QTL analyses have been conducted to identify QTL for various traits in pig production. For example Karlskov-Mortensen et al. reported QTL on chromosomes SSC 1, 4, 9, 10, 13 and 16 affecting fat deposition and lean meat content [5]. For various growth traits QTL were detected on SSC 1, 4, 7 and 8 by de Koning et al. [6]. Several QTL for pH at 45 minutes post mortem have been shown to be located on SSC3, 4, 5, 6, 8, 11, 13 and 17 [7]. Identification of multiple loci associated with one trait suggests an underlying complex genetic architecture. Originally, QTL scans have been mainly performed on experimental crosses between a domestic breed and wild boar or Meishan [8]–[10]. Over the last decade, a number of QTL scans have been carried out on commercial pig line crosses, including Large White, Piétrain, Berkshire and Yorkshire [11], [12].

So far, over 6,800 QTL for 585 different traits have been identified in pigs (PigQTLdb, http://www.animalgenome.org/cgi-bin/QTLdb/SS/index), more than in any other livestock species. The reported QTL were shown to influence meat quality, health, production, reproduction and exterior traits. However, only a few have been further investigated and led to discovery of associated or even causative mutations. These include a single base pair substitution in a non-coding region of *IGF2* on SSC 2 that explains variation in muscle mass and back fat thickness and a nonconservative substitution in the *PRKAG3* gene on SSC 15 having an effect on meat quality [13], [14]. Since most loci explain just a small fraction of the phenotypic variation, the identification of the causative genetic variations underlying QTL remains challenging [15].

The recent development of the PorcineSNP60 BeadChip [16] facilitated by the efforts of the International Swine Genome Sequencing Consortium and Illumina now enables us to perform genome-wide association studies (GWAS) in pigs. Numerous GWAS for quantitative traits have been reported in humans [17], [18] and other domestic animals [19], [20]. However, due to a lack of SNP genotyping arrays, the first GWAS for quantitative traits in pigs have only recently been published. In the last few months several GWAS investigating various traits of commercial or scientific interest in different pig populations were published [21]–[28]. The aim of this study was to identify QTL affecting economically important traits in a Swiss commercial boar line using the PorcineSNP60 BeadChip.

## Results

We used 26 estimated breeding values (EBVs) rather than raw phenotypes for our GWAS. Breeding values have the advantage that they are free of systematic environmental effects on measured phenotypes, as these effects are considered in the statistical model used for the estimation of EBVs. Additionally, they reflect the genetic makeup more accurately because they do not solely rely on own records but include information from all measured relatives. The analyzed traits with available EBVs and key figures of their distribution are shown in Table S1. The EBVs are expressed as a deviation from the mean of a defined group of animals. Therefore the median is close to zero.

We genotyped 192 artificial insemination boars of a commercial line derived from the Swiss Large White breed for 62,163 SNP markers with an average distance of 49 kb. The average call rate per individual was 96.02%. We removed non-informative markers and markers with low call rate. After these quality control steps 186 individuals and 47,045 SNPs remained for the final analysis.

**Figure 1:**
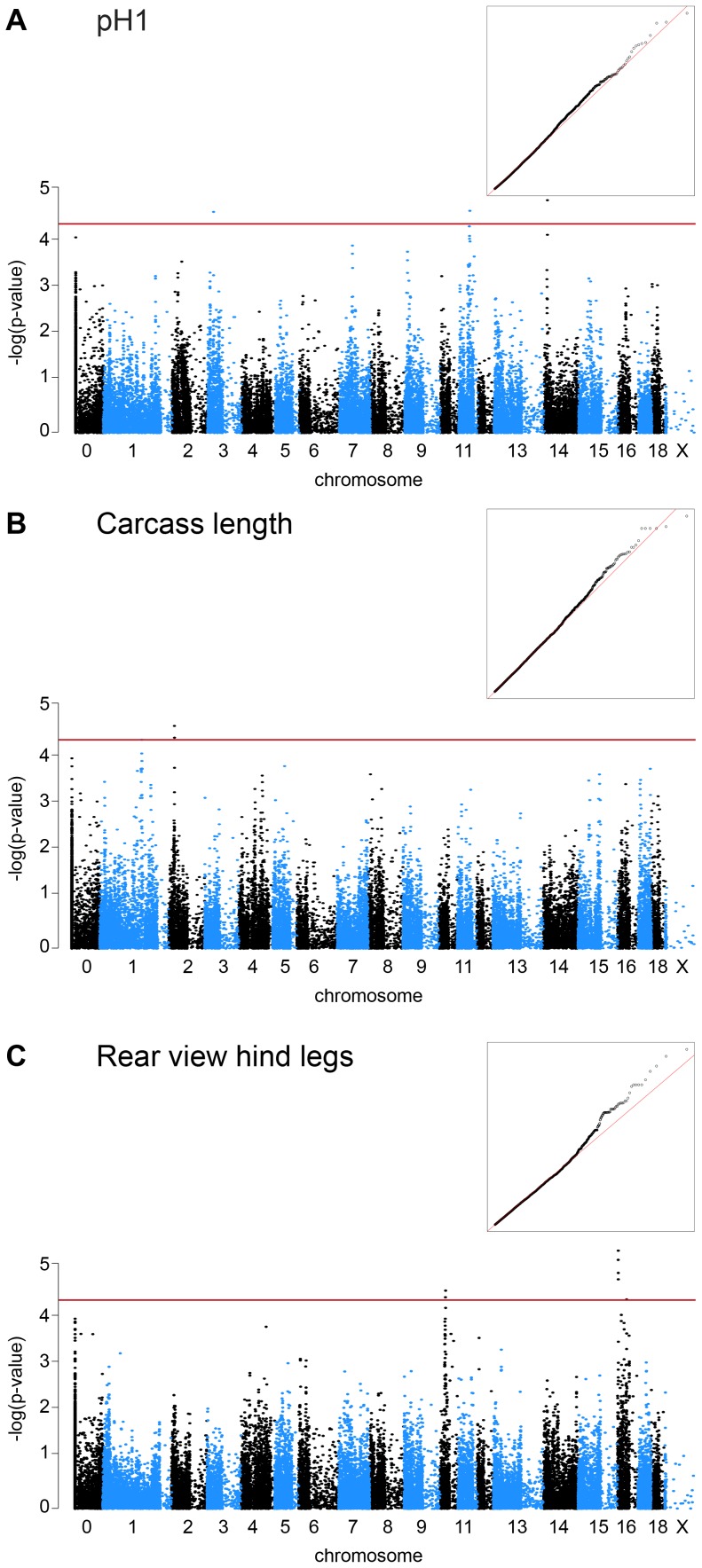
Manhattan plots of genome-wide association studies for EBVs of commercially important traits in pigs. The red lines indicate the significance threshold for moderately significant associations (p = 5×10^−5^). “Chromosome 0” harbors SNP markers that have not yet been mapped to the pig reference genome. The inserted quantile-quantile (QQ) plots show the observed versus expected log p-values. The straight line in the QQ plots indicates the distribution of SNP markers under the null hypothesis and the skew at the right edge indicate that these markers are stronger associated with the traits than it would be expected by chance. Please note that in Figure 1A there are appear to be associated SNPs on SSC 3, 11, and 14. The two associated SNPs on SSC 3 and 11 mapped to these chromosomes in the Sscrofa 9.2 assembly, but are no longer placed on any chromosome in the Sscrofa 10.2 assembly.

We calculated genome-wide pairwise identity-by-state distances and quantified the population stratification (Figure S1). Depending on the analyzed trait the calculated genomic inflation factor varied between 1.15 and 2 indicating that our material was highly stratified. Performing association studies with stratified samples can lead to false positive results, i.e. detected associations can be due to the underlying structure of the population instead of a biologically meaningful association with one or several genes. Therefore, we corrected for the population stratification in our association analysis using the egscore function implemented in GenABEL and by calculating stratified associations within clusters. These corrections in addition to genomic control reduced the genomic inflation factor to reasonable values between 1.00 and 1.01 in all analyses (Figure 1).

We set the p-value thresholds for moderately significant and highly significant associations at 5×10^−5^ and 5×10^−7^, respectively. We did not detect any highly significant associations in our material. However, we detected four QTL with moderately significant associations (Table 1).

**Table 1:** Top allelic association hits in the GWAS for QTL affecting commercially important traits.

Trait	Marker	Chromosome	Position (Sscrofa build 10.2)^b^	Alleles	MAF	p_raw_ a
pH1	ASGA0061594	14	14,730,418	C/T	0.49	1.57×10^−5^
	H3GA0032045	n.d.^b^	n.d.^b^	A/C	0.35	2.59×10^−5^
	ASGA0105130	n.d.^b^	n.d.^b^	C/T	0.49	2.74×10^−5^
carcass length	ASGA0010032	2	42,938,876	A/G	0.38	2.73×10^−5^
	H3GA0006598	2	42,886,909	C/T	0.23	4.76×10^−5^
rear view hind legs	H3GA0045902	16	6,289,550	T/G	0.50	4.99×10^−6^
	ASGA0072056	16	6,198,618	A/G	0.44	7.68×10^−6^
	H3GA0045917	16	6,343,134	C/T	0.40	1.42×10^−5^
	H3GA0045908	16	6,312,026	T/C	0.41	1.96×10^−5^
	ALGA0058443	10	40,670,821	C/A	0.37	3.35×10^−5^
	ALGA0058422	10	39,424,934	A/G	0.44	4.56×10^−5^
	MARC0010334	10	39,538,944	T/C	0.44	4.56×10^−5^
	ALGA0058431	10	39,626,717	C/T	0.44	4.56×10^−5^
	DRGA0010453	10	39,667,084	C/T	0.44	4.56×10^−5^

a p-values were calculated using χ2 tests in an allelic association study.

b The positions of the associated SNPs on the latest version of the pig reference genome were determined by BLAST searches with the flanking sequences of the SNPs as provided by illumina with respect to the Sscrofa 10.2 assembly. Some of the flanking sequences did not give a significant BLAST hit with respect to this genome reference sequence.

For the EBV pH1 we detected a QTL on SSC 14. We determined the positions of the associated markers in the Sscrofa 10.2 assembly of the pig genome. We observed two additional SNPs that are associated with EBV pH1, which are no longer contained in the Sscrofa 10.2 assembly. In the previous Sscrofa 9.2 assembly one of these two SNPs resided on SSC 11, while the other was on SSC 3, and may thus represent potential additional QTL for the EBV pH1.

For the EBV carcass length we observed two closely spaced associated SNPs on SSC 2 (Table 1). The highest significance in our study was observed for a QTL for the EBV rear view hind legs with a p-value after correction for population stratification of 4.99×10^−6^. We observed two QTL supported by multiple closely spaced SNPs for this trait on SSC 10 and SSC 16, respectively (Table 1).

**Figure 2:**
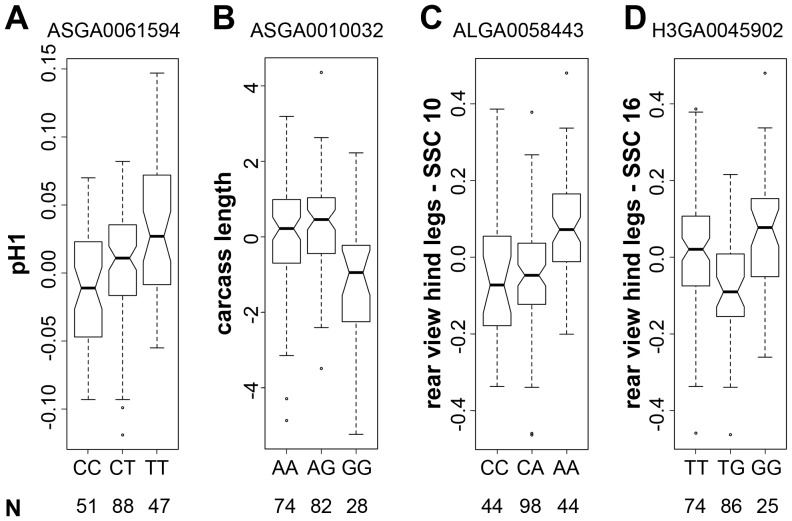
Genotype-phenotype correlations. The animals were grouped according to genotype at the best associated SNPs of each of the four detected QTL. Animal numbers are indicated at the bottom. The call rates for these SNPs were >98.9%. For each of the four QTL the homozygous alternative genotype classes have different phenotypic distributions (p<0.05, Welch's t-test). (A) EBV pH1 distributions with respect to genotype at SNP ASGA0061594 on SSC 14. (B) EBV carcass length in boars with respect to genotype at SNP ASGA0010032 on SSC 2. (C) EBV rear view hind legs distributions in Swiss Large White boars with respect to genotype at SNP ALGA0058443 on SSC 10. (D) EBV rear view hind legs distributions in Swiss Large White boars with respect to genotype at SNP H3GA0045902 on SSC 16.

We grouped the animals according to genotype at the best-associated SNPs for the four detected QTL and analyzed their phenotype distribution (Figure 2). The QTL for EBV pH1 showed a largely additive effect. In contrast, the genotypes at the QTL for the EBV carcass length and the QTL on SSC 10 for EBV rear view hind legs indicated a recessive effect of the variant allele. Finally, at the QTL on SSC 16 for EBV rear view hind legs, heterozygous animals showed EBVs that are outside of the range of animals with the two alternative homozygous genotypes (overdominance).

## Discussion

In this study we identified four QTL with moderate significance in a Swiss commercial pig population. We think that the relatively low number of detected QTL is primarily due to the small number of animals used in this study. The population substructure with different subclusters in our animal cohort (Figure S1) required correction of the p-values for the effects of the population stratification, which also negatively affected the power of the GWAS. The limited power of our study design is reflected by the fact that we only found QTL with very even allele distributions. The best-associated SNPs at the four detected QTL had MAFs between 0.37 and 0.50. Considering all associated SNPs, the lowest MAF was 0.23 at marker H3GA0006598 on SSC 2. The detection of QTL caused by rare alleles of the same effect size would have required larger animal numbers.

For three of the four detected QTL, similar QTL have previously been identified at roughly the same locations in other pig populations. Lee et al. reported a QTL for carcass length in an experimental Meishan×wild boar family on SSC 2 at 20.8 cM [29]. Additionally, Evans et al. identified a QTL for carcass length on SSC 2 in the region of 0–10 cM in a Landrace population [30]. A QTL for “rear upright legs”, which might be a correlated trait to the rear view hind leg score of our analysis, was identified at 43 Mb on SSC 10 [31] This QTL was identified by genome-wide association study in commercial pigs from Large White or Large White×Landrace crosses. Another study identified a QTL for rear leg score in a Japanese Landrace population at 82 cM on SSC 10 [32]. Finally, Lee et al. reported QTLs for the trait “back legs” in a Large White×Meishan crossbred population on SSC 10 and SSC 16 at 126 cM and 10 cM, respectively [33]. It is very difficult to evaluate whether these reported QTL are really the same QTL that we found as QTL from linkage analyses were typically mapped with very low resolution and have very large confidence intervals. If QTL are independently discovered in different populations, this suggests that they may indeed be due to a biologically relevant genetic variation rather than to confounding effects such as e.g. population stratification artifacts.

So far, we have no knowledge of a study concerning the trait pH one hour post mortem. There are several studies about the pH 45 minutes post mortem which closely correlates with the trait used in our analysis (PigQTLdb, http://www.animalgenome.org/cgi-bin/QTLdb/SS/index). However, a QTL for pH 45 minutes post mortem has not been reported on SSC 14. Given the moderate significance of our QTL, a replication study with independent animals would be desirable to confirm the results. Nonetheless, our findings offer a chance to unravel new QTL that contribute to the meat maturation.

The QTL for EBV pH1 detected in our study shows a largely additive effect (Fig. 2A) whereas the QTL for EBV carcass length and EBV rear view hind legs on SSC 10 indicate a recessive effect of the variant allele (Fig. 2B & C). This could be either due to coding variants or to regulatory variants that change the quantitative expression levels of the causative genes. On the other hand, the QTL for EBV rear view hind legs on SSC 16 does not show a simple additive effect (Fig. 2D). For this QTL, animals being heterozygous at the best associated SNP show more extreme phenotypic levels than animals with either homozygous genotype. It seems unlikely that such an overdominance effect can be caused by simple quantitative differences in mRNA expression of the underlying genes. One possible explanation would be provided by non-synonymous variants in genes encoding oligomeric proteins. In such a scenario, it is feasible that oligomers of truly identical proteins (in homozygous animals) have very different properties than oligomers of allelic variants (in heterozygous animals).

Our study provides another example of the usefulness of the PorcineSNP60 BeadChip for genome-wide association studies in pigs. This tool allows the detection of QTL for commercially important traits in pigs. However, it must also be noted that due to the imperfect pig reference genome assembly, the exact genome positions of many markers on this tool are not clear. We observed very significant shifts of some associated markers between the Sscrofa9.2 and the Sscrofa10.2 assembly. Consequently, the marker spacing may also be expected to be somewhat irregular. Thus, it is quite possible that some real QTL might have been missed due to insufficient marker coverage of the PorcineSNP60 BeadChip. With rapidly increasing genomic resources for the pig, it may be expected that improved genotyping tools will soon become available.

In conclusion, we have mapped four QTL by genome-wide association mapping in Swiss commercial pigs. Three of these QTL coincide with previously detected QTL for similar traits in other independent pig populations while the QTL for EBV pH1 on SSC 14 is described for the first time.

## Materials and Methods

### Animals and phenotypic data

We obtained previously archived tissue samples of 192 artificial insemination boars from the commercial Premo® line, which is derived from the Swiss Large White breed. The animals were from the breeding company SUISAG (www.suisag.ch) and born between 2004 and 2009. All animals underwent a performance and progeny test according to the test scheme of SUISAG [34]. We obtained 26 corresponding estimated breeding values (EBV) for different pig production traits and used them as phenotypes in our analysis (Table S1). The EBVs were routinely estimated by SUISAG in 3 separate analyses using multiple trait animal models and BLUP [35]. For exterior traits linear description scores by trained technicians on animals tested in the central testing station Sempach and on-farm tested selection candidates were considered. The trait rear view hind legs describes the hind legs from an extreme X- (score 1) to an extreme O- form (score 7). Carcass length was measured in centimeters from the cranial edge of the first cervical vertebra to the cranial edge of the pelvic bone 24 h post mortem in all station tested pigs. The estimation for production traits of field tested crossbred progeny of artificial insemination sires were included in addition to the animals scored for exterior traits. pH1 was measured 1 hour post mortem at the *musculus longissimus dorsi* in all station tested pigs. Mating and litter records obtained from herdbook farms were used for the estimation of breeding values for reproduction traits. There were less animals with EBVs on reproduction traits, as artificial insemination sires need litter records of daughters to reach an acceptable accuracy.

### SNP array genotyping

We isolated genomic DNA from tissue samples with the Nucleon Bacc2 kit (GE Healthcare) according to the manufacturer's protocol. DNA samples with a ratio of A260/280 higher than 1.8 and a concentration of approximately 50 ng/µl were genotyped at the Leibniz Institute for Farm Animal Biology, Dummerstorf, Germany using the Illumina Porcine60SNP BeadChip containing 62,163 markers. We used BLASTN to determine the positions of the markers in the Sscrofa 10.2 genome reference assembly of the pig.

### Quality control and genome-wide association analyses

We analyzed the data with the GenABEL package [36] in the R environment. Initially, we removed all individuals with a call rate <95% and checked the dataset for replicates and gender mismatch. We excluded markers strongly deviating from Hardy-Weinberg equilibrium (p<0.0001), markers having a call rate <95%, and markers with a minor allele frequency of <5%. After these quality control steps 186 individuals and 47,045 SNPs remained for the analysis.

We calculated genome-wide pairwise identity-by-state (IBS) distances to measure population stratification as we used samples of animals with expected diverse genetics. Based on genetic distances between individuals projected into two-dimensional space using multidimensional scaling we grouped the animals into three subpopulations. Additionally, the procedure egscore which uses principal component analysis [37] was used to correct for population stratification in the dataset. We performed allelic genome-wide association analyses for all 26 EBVs. We considered p-values<5×10^−7^ as indicative for strong evidence of association and p-values between 5×10^−5^ and 5×10^−7^ as indicative for moderate evidence of association according to the recommendation of the Wellcome Trust Case Control Consortium [38].

## Supporting Information

Figure S1 **Multidimensional scaling (MDS) plot showing the genomic kinship between the analyzed animals.** This plot visualizes the overall genetic distances between the boars based on 2,000 markers randomly selected out of the total of 47,045 SNP markers. We grouped the animals into three subpopulations based on genetic distances between individuals.
(PDF)

Table S1 **Estimated breeding values of genotyped material with their median and distribution.**
(PDF)
