# Supplementary material for: A Genome-Wide Association Study to Detect QTL for Commercially Important Traits in Swiss Large White Boars
Source: PLoS One. 2013 Feb 5;8(2):e55951. doi: 10.1371/journal.pone.0055951 (PMC3564845; doi:10.1371/journal.pone.0055951)
Supplement: Figure S1 — Multidimensional scaling (MDS) plot showing the genomic kinship between the analyzed animals. This plot visualizes the overall genetic distances between the boars based on 2,000 markers randomly selected out of the total of 47,045 SNP markers. We grouped the animals into three subpopulations based on genetic distances between individuals. (PDF) [file pone.0055951.s001.pdf]

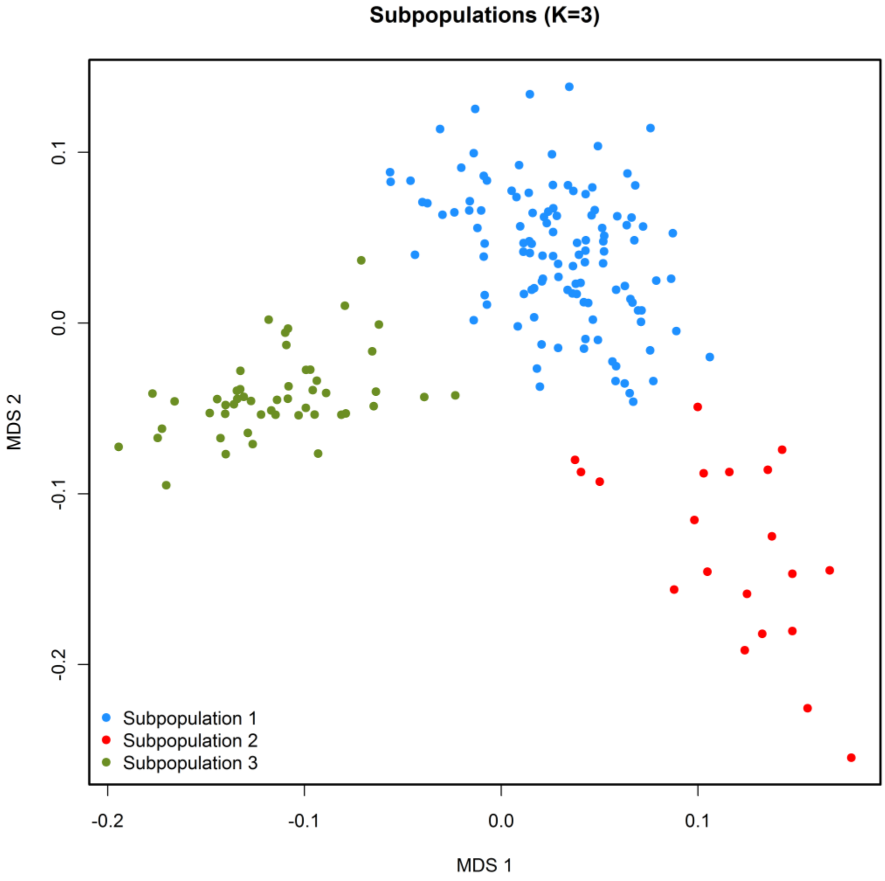

Figure S1. Multidimensional scaling (MDS) plot showing the genomic kinship between the analyzed animals. This plot visualizes the overall genetic distances between the boars based on 2,000 markers randomly selected out of the total of 47,045 SNP markers. We grouped the animals into three subpopulations based on genetic distances between individuals.
