# Supplementary material for: A Genome-Wide Association Study to Detect QTL for Commercially Important Traits in Swiss Large White Boars
Source: PLoS One. 2013 Feb 5;8(2):e55951. doi: 10.1371/journal.pone.0055951 (PMC3564845; doi:10.1371/journal.pone.0055951)
Supplement: Table S1 — Estimated breeding values of genotyped material with their median and distribution. (PDF) [file pone.0055951.s002.pdf]

Table S1. Estimated Breeding values of genotyped material with their median and distribution

EBV	Units	N	Median	95% LCL	95% UCL	Min	Max
Production							
daily gain on test	g/day	192	20.1547	11.6392	27.1212	-71.7338	147.3842
feed conversion	kg/kg	192	-0.0471	-0.0646	-0.0336	-0.2246	0.1884
muscle cross section surface	cm^2	192	0.1899	-0.0296	0.5034	-6.9906	4.7544
intramuscular fat	%	192	0.0244	-0.0581	0.0799	-0.7051	0.8029
pH1 post mortem	pH unit	192	0.0120	0.0030	0.0190	-0.1190	0.1470
pigmentation	unit	192	-0.0079	-0.0164	0.0016	-0.1464	0.1456
reflectance	Unigalvo unit	192	0.1528	-0.0337	0.3383	-2.5567	2.2353
drip loss	%	192	-0.0643	-0.1603	0.0857	-1.0463	1.5417
average daily gain on farm	g/day	192	5.6959	2.8704	9.3034	-38.2676	73.2584
ultrasound backfat thickness	mm	192	-0.3315	-0.4775	-0.1995	-3.3255	2.6485
average daily gain of slaughter pigs	g/day	192	7.5127	5.0337	10.7007	-40.2963	80.5897
lean meat content	%	192	0.3811	0.0486	0.6096	-3.0364	3.4586
Exterieur							
number of teats	number	192	0.0214	-0.1316	0.0944	-1.3266	1.5054
number of inverted teats	number	192	-0.0207	-0.0332	-0.0082	-0.2602	0.3168
rear view hind legs	scale 1-7	192	-0.0014	-0.0389	0.0331	-0.4629	0.4801
side view hind legs	scale 1-7	192	0.0008	-0.0272	0.0338	-0.3472	0.3498
side view pastern	scale 1-7	192	-0.0076	-0.0531	0.0459	-0.7001	0.6199
size of inner claw	scale 1-7	192	0.0078	-0.0352	0.0368	-0.4602	0.3658
side view forelegs	scale 1-7	192	-0.0126	-0.0271	0.0039	-0.2561	0.5549
number of bursae with subcutaneous liquid	number	192	-0.0061	-0.0956	0.0714	-1.4116	1.4834
gait	scale 4-7	192	-0.0114	-0.0274	0.0016	-0.2984	0.3086
carcass length	cm	192	0.0980	-0.1625	0.3365	-5.2475	4.3535
Reproduction							
number born alive	number	108	-0.1828	-0.5133	-0.0993	-1.6463	3.0897
proportion of underweight piglets	%	108	-0.1549	-0.4309	0.0721	-4.8019	5.8151
survival rate piglets	%	108	-0.0878	-0.6633	0.5207	-4.8983	5.3567
interval weaning to mating	day	108	-0.0464	-0.2924	0.0816	-2.0844	2.9266
